# Role of C–N Configurations in the Photoluminescence of Graphene Quantum Dots Synthesized by a Hydrothermal Route

**DOI:** 10.1038/srep21042

**Published:** 2016-02-15

**Authors:** Fitri Aulia Permatasari, Akfiny Hasdi Aimon, Ferry Iskandar, Takashi Ogi, Kikuo Okuyama

**Affiliations:** 1 Department of Physics, Faculty of Mathematics and Natural Sciences, Institut Teknologi Bandung, Bandung 40132, Indonesia; 2 Research Center for Nanoscience and Nanotechnology, Institut Teknologi Bandung, Bandung 40132, Indonesia; 3 Department of Chemical Engineering, Graduate School of Engineering, Hiroshima University, Higashi-Hiroshima 739-8527, Japan

## Abstract

Graphene quantum dots (GQDs) containing N atoms were successfully synthesized using a facile, inexpensive, and environmentally friendly hydrothermal reaction of urea and citric acid, and the effect of the GQDs’ C–N configurations on their photoluminescence (PL) properties were investigated. High-resolution transmission electron microscopy (HR-TEM) images confirmed that the dots were spherical, with an average diameter of 2.17 nm. X-ray photoelectron spectroscopy (XPS) analysis indicated that the C–N configurations of the GQDs substantially affected their PL intensity. Increased PL intensity was obtained in areas with greater percentages of pyridinic-N and lower percentages of pyrrolic-N. This enhanced PL was attributed to delocalized π electrons from pyridinic-N contributing to the C system of the GQDs. On the basis of energy electron loss spectroscopy (EELS) and UV-Vis spectroscopy analyses, we propose a PL mechanism for hydrothermally synthesized GQDs.

As emerging carbon nanomaterials, graphene quantum dots (GQDs) have received enormous attention because of their unique chemical, electronic, and optical properties^1^,^2^,^3^. Various approaches developed to prepare GQDs include acidic oxidation, hydrothermal synthesis, microwave synthesis, electrochemical synthesis, oxygen plasma etching, catalysed cage-opening, pyrolysis, and exfoliation methods^4^,^5^,^6^,^7^,^8^,^9^. Moreover, numerous works concerning the mechanism of GQD formation via various simple synthesis methods have been reported^1^,^10^,^11^. Tang *et al.* first reported a simple method for the preparation of GQDs by microwave-assisted hydrothermal synthesis using glucose as a precursor^12^. On the basis of their experimental results, they deduced that the formation steps of the GQDs were nucleation and surface growth followed by the development of a self-passivation layer. Dong *et al.* reported a pyrolysis method to selectively prepare GQDs and graphene oxide (GO) by controlling the carbonization of citric acid^10^. Using of this method, they prepared GQDs and GO by directly pyrolysing citric acid at 200 °C. The liquid sample changed from colourless to pale-yellow and then to orange after 30 min; a black solid was finally obtained after ~120 min. Qu *et al.* investigated the effects of atom doping on GQDs (e.g., Na-doped and N-doped GQDs) prepared from various precursors using the hydrothermal method^1^. They reported that the greatest PL performance was achieved with GQDs that were synthesized from citric acid and urea as precursors.

Our group has reported a similar synthesis of GQDs by reacting citric acid and urea via a hydrothermal method^11^. However, the hydrothermal reactor system was modified to allow the sample to be extracted at a selected elapsed time during the synthesis process. Consistent with the results of Dong *et al.*^10^ the prepared samples exhibited colour changes as the reaction between the citric acid and urea progressed. In our previous report, the transient nature of GQD formation was proposed on the basis of the results of nuclear magnetic resonance (NMR) and Fourier transform infrared (FTIR) analyses. The formation of GQD began with the reaction of citric acid and urea into citric acid amide. Further heating of the sample under hydrothermal conditions led to dehydration and deamination among the carboxyl, hydroxyl, and amino groups of the intermolecular compounds, which resulted in the self-assembly of the citric acid amide into a nanosheet structure. The luminescence effect observed in the obtained GQDs was attributed to the condensate of the citric acid amide. The sample prepared in the absence of the urea precursor exhibited no luminescence, indicating that nitrogen from the urea played an important role in the observed photoluminescence (PL) effect. Similar results have been reported by several researchers who observed that nitrogen improves the PL of GQDs^1^,^9^. Because the C–N configurations, i.e., pyrrolic-N, graphitic-N, and pyridinic-N, affected the electronic structure of GQDs^13^,^14^,^15^, elucidating the role of N configurations in GQDs is important for understanding their PL mechanism. To the best of our knowledge, the literature contains no reports regarding the role of these three C–N configurations or their relationship to the PL intensity of GQDs derived from citric acid and urea precursors with hydrothermal method. Therefore, in this work, we used transmission electron microscopy (TEM), X-ray photoelectron spectroscopy (XPS), UV-Vis spectroscopy, and energy electron loss spectroscopy (EELS) to elucidate the role of the C–N configuration, i.e., pyrrolic-N, graphitic-N, and pyridinic-N, on the PL effect in GQDs. On the basis of these results, we also propose a GQD PL mechanism that accounts for the effects of the C–N configurations.

## Results

**Figure 1:**
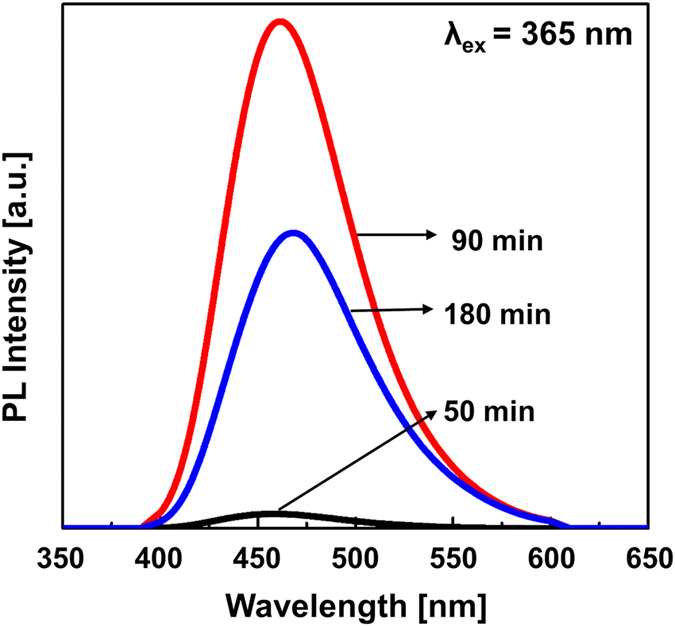
PL intensity of the GQD samples under an excitation wavelength of 365 nm.

The PL spectra of the samples under excitation at 365 nm are shown in Fig. 1. The spectra of all samples showed an emission peak at approximately 460 nm, that is, a blue emission. The PL intensity of the samples changed depending on the elapsed time of the synthesis process. The PL intensity was substantially increased after 50 min and reached a maximum intensity at 90 min of hydrothermal reaction.

### Morphology and Bandgap Energy Approximation of GQDs

**Figure 2:**
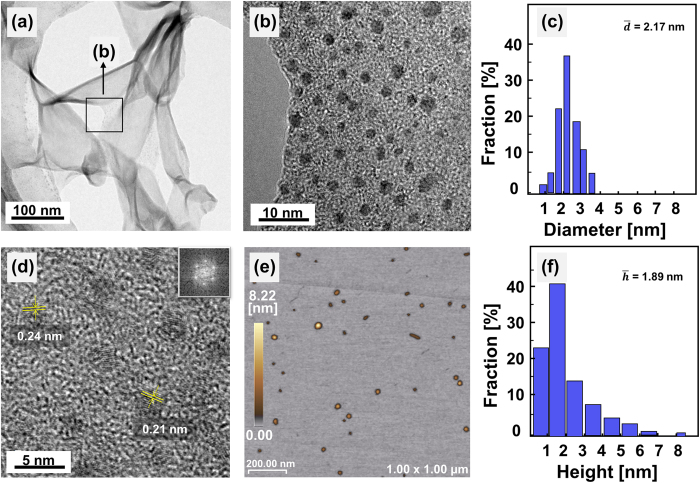
TEM images of the sample obtained after 90 min: (a) low magnification; (b) high magnification; (c) size distribution of the dots; (d) HR-TEM image. The inset shows the 2D FFT image corresponding to the selected area. (**e**) AFM image. (**f**) Height profile of the selected area in (**e**).

Figure 2 shows TEM images of the sample obtained at 90 min. Low-magnification TEM images (Fig. 2a) revealed that the sample consisted of sheets. Furthermore, high-magnification TEM (Fig. 2b) confirmed that some areas of the sheets consisted of dots with spherical shapes. By measuring hundreds of dots, we determined that the dots were approximately 1–3.5 nm in size, with an average diameter of 2.17 nm (Fig. 2c). In addition, high-resolution TEM images (Fig. 2d) show that the dots exhibited good crystallinity, with lattice fringes of 0.21 nm and 0.24 nm, which correspond to the 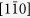 plane of graphene^2^ and the 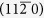 lattice of graphite^1^, respectively. Finally, the fast Fourier transform (FFT) results obtained for a selected dot (as shown in the corner of Fig. 2d) indicate that the dots exhibited a hexagonal structure. Atomic force microscopy (AFM) imaging (Fig. 2e) revealed a typical topographic height of 0–8.22 nm, with an average height of 1.89 nm (Fig. 2f); these results suggest that the average particle size was 1.89 nm, which is very similar to the average size observed by TEM (2.17 nm). This average size also suggests that the GQDs consist of 5–6 graphene layers on average.

Because quantum confinement affects the bandgap energy of particles less than 10 nm in size, the effective mass approximation (EMA) model in Equation (1) was used to estimate the bandgap of the prepared sample^16^.


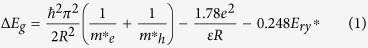


The calculation was simplified by assuming the prepared GQD was epitaxial graphene. Whereas the gap energy for graphene without the quantum confinement effect is equal to zero^17^, the effective electron mass is 0.19*m*_*o*_^18^ and the effective hole mass is 0.25*m*_*o*_, where *m*_*o*_is the free electron mass. Because of the extreme value of the dielectric constant of graphene^19^,^20^,^21^,^22^, the second and third terms in Equation (1) are negligible. Therefore, according to this equation, a GQD 2.17 nm in diameter has an energy gap of approximately 2.96 eV. Converting this gap energy into an emission wavelength gives an emission wavelength of approximately 430 nm. This value is similar to the emission peak wavelength observed in the PL spectrum of the sample (~460 nm).

### C–N Configurations of the GQDs

A general overview of the chemical composition of the samples was obtained using XPS full scans. Three peaks at ~288.8 eV, ~399.5 eV, and ~531.5 eV were observed in all of the XPS full-scan spectra (see Supplementary Fig. S2); theses peaks can be assigned to C1s, N1s, and O1s, respectively. No other peaks were present, which means that the sample surface consisted of only three elements—C, N, and O—without any contaminants. Several previous reports suggest that these three elements are the constituents of GQDs^11^,^23^,^24^.

**Figure 3:**
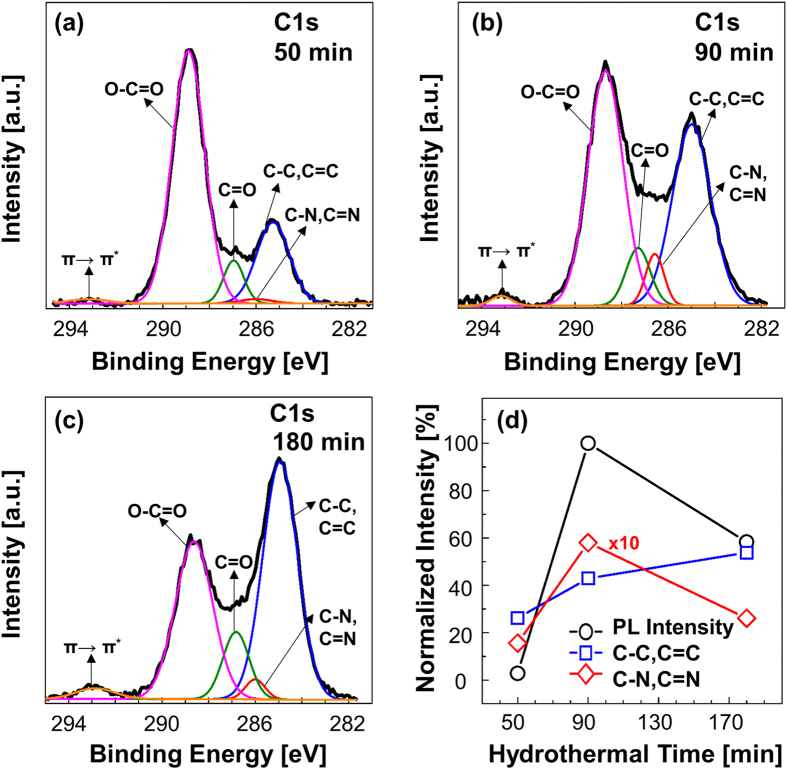
C1s and deconvoluted spectra of the samples obtained after (a) 51 min; (b) 90 min; (c) 180 min.

To further elucidate the contribution of the C–N configuration to the PL mechanism, detailed C and N XPS scans were conducted. The detailed C1s spectrum is shown in Fig. 3. The spectrum consisted of two main peaks at ~285 eV and ~289 eV, which correspond to pristine C and O functional groups, respectively. To further elucidate the correlation between the PL intensity and the C1s XPS spectrum, we deconvoluted the spectrum into six separate spectra on the basis of the FTIR results from a previous report^11^. These deconvoluted spectra included a C–C, C=C peak with a binding energy of ~284 eV^24^,^25^,^26^, a C–N, C=N peak with a binding energy of ~286 eV^24^,^25^,^26^, a C=O (carbonyl group) peak with a binding energy of ~287 eV, an O–C=O (carboxylate group) peak with a binding energy of ~288 eV^15^,^27^, and a shake-up peak with a binding energy of ~292 eV^3^,^28^,^29^. The shake-up peak is a characteristic of conjugated systems or aromatic groups^30^. Moreover, the presence of the C–C, C=C, and shake-up peaks confirmed the hexagonal structure of the graphene^11^,^24^,^25^,^26^. The intensity of the shake-up peak increased with increasing reaction time, indicating that the quality of the GQDs was improved by a longer hydrothermal reaction time. The percentage contributions of the C–C and C=C bonding and C–N and C=N bonding peak areas to the PL intensity are shown in Fig. 3d. The percentage areas of the C–C and C=C bonding increased with increasing the reaction time, consistent with an increase in intensity of the shake-up peak. Moreover, the trend of the percentage areas of the C–N and C=N bonding was consistent with the trend of PL intensity. These results indicate that C–N configurations play an important role in determining the PL intensity of the GQDs.

**Figure 4:**
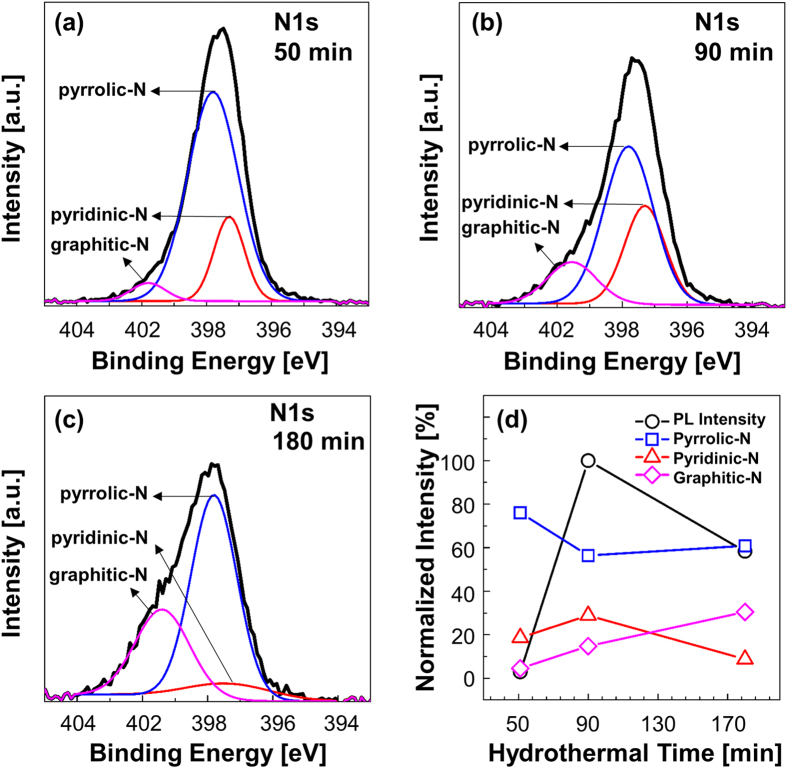
N1s and deconvoluted spectra of the samples obtained after (a) 51 min; (b) 90 min; (c) 180 min. (**d**) Dependence of the PL intensity on the N-configuration composition.

Peak deconvolution analysis of the N1s XPS spectra was also conducted, as shown in Fig. 4. The spectra were deconvoluted into three peaks: pyridinic-N (398.1–399.3 eV), pyrrolic-N (399.8–401.2 eV), and graphitic-N (401.1–402.7 eV)^13^,^23^. The results show that, except for the sample at 180 min of hydrothermal time, the C–N configuration was dominated by pyrrolic-N and pyridinic-N. These results agree with the previous results obtained for GQDs synthesized from citric acid and urea precursors^31^,^32^. The trends in the percentage areas of the deconvoluted N1s peaks also show that the increasing PL intensity of the sample at 90 min was caused by a decrease in the amount of pyrrolic-N and an increase in the amount of pyridinic-N in the GQDs. This result is attributable to pyridinic-N contributing one electron to the π system across the C atoms, which is known as a delocalized electron^2^,^10^. The diverse wavefunction of such a delocalized electron may lead to a narrower energy gap between π and π^*^, which would increase the probability of electron excitation for the same excitation energy. By contrast, pyrrolic-N (pentagonal structure with sp^3^ hybridization) causes a high non-radiative transition^33^. As a result, the PL intensity of the sample with 180 min reaction time was decreased by the decrease in the amount of pyridinic-N and increase in the amount of pyrrolic-N in this sample. In addition, the XPS spectrum of the sample obtained at 180 min exhibited a high proportion of graphitic-N, possibly because of the high stability of graphitic-N during the high-temperature synthesis^34^.

### Optical Transition of GQDs

The UV-Vis absorption spectrum of the GQD sample obtained at 90 min (see Supplementary Fig. S3) exhibited a broad single peak at approximately 340 nm. This spectrum differs from that of graphene oxide dots, which does not show a main peak^5^,^10^. The UV-Vis absorption spectrum showed two possible transitions for the GQDs: a π → π^*^ transition of sp^2^ C at 292 nm^35^ and an n → π^*^ transition at 340 nm^1^,^36^. These two transitions provide information about the mechanism of PL in the GQDs.

**Figure 5:**
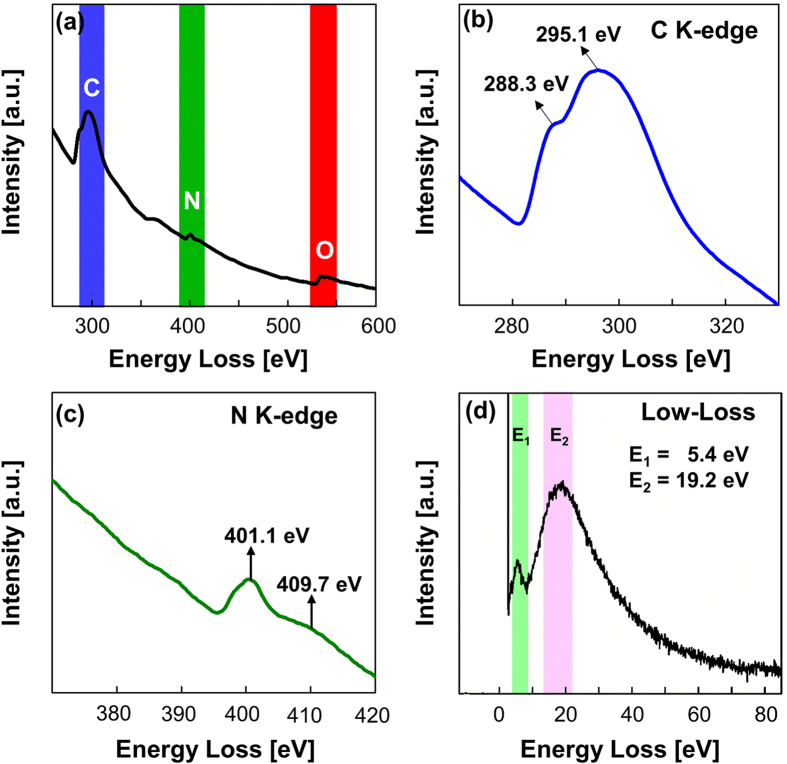
EELS results for GQDs obtained after 90 min: (a) full-scan K-edge; (b) C K-edge; (c) N K-edge; (d) low-loss spectrum.

Figure 5 shows the results of the EELS measurements of the sample obtained at 90 min. The EELS spectrum exhibited main peaks at 295.1 eV, 401.1 eV, and 534.5 eV, which are attributed to the K-edge of C^37^,^38^, N^2^, and O^28^, respectively. The presence of these peaks agrees with the XPS results, which indicated the presence of only C, N, and O. The C K-edge spectrum, as shown in Fig. 5b, exhibited peaks at 288.3 eV and 295.1 eV, which correspond to 1s → π^*^ and 1s → σ^*^ transitions, respectively. These peaks are relatively higher in energy loss compared to the C K-edge peaks of graphite (1s → π^*^ at 285 eV and 1s → σ^*^ at 291 eV)^39^,^40^. Moreover, the shape of the π^*^ peak indicates pure sp^2^ hybridization, whereas the broadness of the σ^*^ peak indicates a contribution from the C–N configurations instead of the C–C configurations^26^. However, the N K-edge spectrum (Fig. 5c) shows two weak peaks at 401.1 eV and 409.7 eV, which are attributable to 1s → π^*^ and 1s → σ^*^ at C=N, respectively^41^,^42^. Similar to the C K-edge spectrum, the O K-edge spectrum (see Supplementary Fig. S4) exhibits peaks at 534.5 eV and 540.2 eV that are attributed to 1s → π^*^ and 1s → σ^*^ at C=O, respectively^43^,^44^.

Figure 5d shows the low-loss spectrum of the sample obtained at 90 min. The spectrum exhibits two peaks at 5.4 eV and 19.2 eV. The first peak is attributed to surface plasmon π^*^, which is relatively lower in energy than that of graphite (i.e., ~7 eV) but greater than that of single-layer graphene oxide (i.e., ~5 eV)^38^,^39^,^45^. The second peak is attributed to the surface plasmon π^*^+σ^*^. According to Qu *et al.*^46^, an oxygen-free pure graphene sheet exhibits a surface plasmon π^*^+σ^*^ energy of approximately 22.7 eV that shifts in relation to the number of graphene layers. Eberlin *et al.* determined surface plasmon π^*^+σ^*^ energies of approximately 14.6, 18.0, and 26.0 eV for one, five, and ten layers of graphene, respectively^45^,^46^. By extrapolating these values, we determined that the GQDs obtained at 90 min contained approximately 5–6 layers of graphene, consistent with the layer estimation based on the AFM image (Fig. 2e,f).

## Discussion

**Figure 6:**
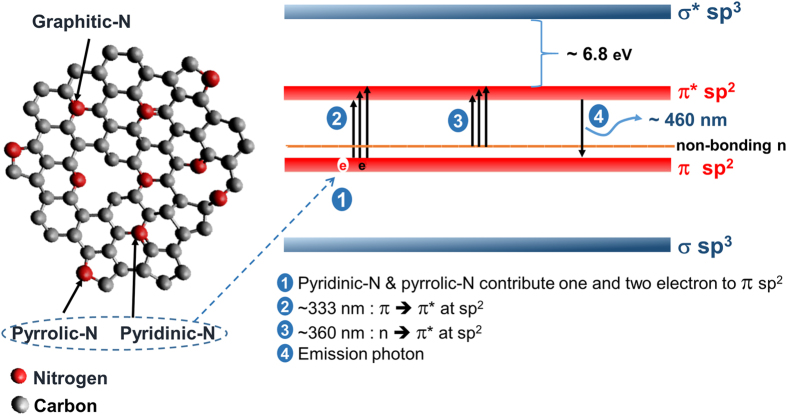
(**a**) C–N configurations in the GQDs. (**b**) Illustration of the PL mechanism in the GQD based on experimental results.

The proposed PL mechanism for GQDs synthesized using the present process is illustrated in Fig. 6. The emission mechanism of the GQDs depends on sp^2^ clusters that are isolated within an sp^3^ C–O matrix, as proposed by Eda *et al.* for the emission mechanism in graphene oxide^47^. Additionally, the C–N configurations (pyridinic-N and pyrrolic-N) contribute one or two electrons, respectively, to *π* at GQD sp^2^ as delocalized electrons. The delocalized electron is excited to π^*^ bonds at sp^2^ C with the *π* electron of the sp^2^ C. Therefore, the excited electrons recombine with holes in the *π* bonds at GQD sp^2^. As a result, PL is emitted from the sample at an emission wavelength of approximately 460 nm. The presence of pyrrolic-N as a pentagonal structure in the GQD leads to non-radiative transition of the excited electron.

Another PL mechanism of GQDs involves electrons undergoing π → π^*^ transitions at C=C, π → π^*^ transitions at C=O, and π → π^*^ transitions at C=N when illuminated by light of a certain energy. The excited electrons can be deactivated by direct recombination or by undergoing intersystem crossing (C π^*^ →  N π^*^ or N π^*^ →  O π^*^) followed by vibration relaxation and radiative recombination. Tang *et al.*^2^ considered a similar mechanism. The other possibility for electron deactivation in GQDs involves the surface functional groups with various energy levels. Radiative recombination induces excitation-wavelength-dependent PL through a series of emissive traps^39^. However, both of these alternate PL mechanisms are unlikely because, as evident in the UV-Vis spectra, no peak associated with the transition of the intersystem crossing and the functional surface.

## Conclusions

The photoluminescence effect in GQDs was systematically studied. HR-TEM images showed that particular areas of the synthesized sheets consisted of dot particles with spherical shapes. FFT results for a selected dot confirmed a hexagonal structure. The dots were 1–3.5 nm in size, with an average diameter of 2.17 nm. The appearance of a shake-up peak and C–C and C=C bonding peaks in the C1s XPS spectra confirmed that the quality of the graphene structure improved with increasing hydrothermal reaction time. Moreover, the XPS results revealed that C–N species substantially contributed to the PL intensity of the GQDs. Increased PL intensity was obtained for samples with greater proportions of pyridinic-N and lower proportions of pyrrolic-N. Whereas each pyridinic-N contributes one electron to the GQD and, thus, increases the PL intensity, each pyrrolic-N causes a non-radiative transition of the excited electron. The UV-Vis results showed that the PL effect originated from the electron transition from C-sp^2^ hybridization. Finally, EELS analysis indicated that our GQDs consisted of 5–6 layers of graphene.

## Methods

### Experimental

The graphene quantum dots (GQDs) were synthesized using a hydrothermal route with citric acid and urea as the precursors. The details of the synthesis have been explained elsewhere^11^. In brief, citric acid (C_6_H_8_O_7_, Sigma-Aldrich) and urea ((NH_2_)_2_CO, Sigma-Aldrich) were used as carbon and nitrogen sources, respectively. A precursor solution was prepared by dissolving 75 mg urea and 40 mg citric acid in 100 mL pure water at room temperature under stirring for 5 min. The molarities of C and N in the solution were fixed at 0.0042 mol/L and 0.25 mol/L, respectively. The precursor solution was heated in a stainless steel autoclave with an inner Teflon liner for 180 min at 0.8–1.0 MPa under a specific temperature profile (see Supplementary Fig. S1). The hydrothermal system was modified to allow extraction of the sample solution from the autoclave after a certain elapsed reaction time. To investigate the transformation of the C–N configurations in the GQDs, samples were collected from the autoclave at elapsed reaction times of 50, 90, and 180 min during the hydrothermal synthesis.

### Characterization

The PL intensity of the samples was measured using a spectrofluorophotometer (RF 5300PC, Shimadzu Corp., Kyoto, Japan) equipped with a xenon lamp. The sample obtained after 90 min of elapsed reaction time was characterized by AFM (SPM 9700, Shimadzu, Japan) in dynamic mode. The sample was also characterized by HR-TEM on an electron microscope (JEM-3000F, JEOL, Tokyo, Japan) equipped with an electron energy loss spectroscopy (EELS) apparatus to investigate the morphology, atomic composition, and transition spectra of the sample. The EELS spectra were analysed using the Gatan Digital Micrograph software. The absorption spectra of the sample was measured using UV-Vis spectroscopy (UV-2450, Shimadzu Corp.).

All samples were characterized by XPS (Quantera II, PHI Corp., Japan) to investigate their atomic composition and C–N configurations. The atomic composition of the samples was determined from the percentage peak areas of the C1s, N1s, and O1s peaks of the full-scan XPS spectra. The XPS data were analysed using the CasaXPS software by applying a Shirley background with relative sensitivity factors (RSFs) of 1.00, 1.80, and 2.93 for C1s, N1s, and O1s, respectively. To investigate the C–N configurations in the samples, the C1s and N1s spectra were deconvoluted used the Gaussian-Lorentzian function.

## Additional Information

**How to cite this article**: Permatasari, F. A. *et al.* Role of C-N Configurations in the Photoluminescence of Graphene Quantum Dots Synthesized by a Hydrothermal Route. *Sci. Rep.* **6**, 21042; doi: 10.1038/srep21042 (2016).

## Supplementary Material

Supplementary Information
