# Supplementary material for: Role of C–N Configurations in the Photoluminescence of Graphene Quantum Dots Synthesized by a Hydrothermal Route
Source: Sci Rep. 2016 Feb 15;6:21042. doi: 10.1038/srep21042 (PMC4753454; doi:10.1038/srep21042)
Supplement: Supplementary Information [file srep21042-s1.doc]

**Supplementary Information**

**Role of C–N Configurations in the Photoluminescence of Graphene Quantum Dots Synthesized by a Hydrothermal Route**

Fitri Aulia Permatasari^1^, Akfiny Hasdi Aimon^1^, Ferry Iskandar^1,2^,*,
Takashi Ogi^3^, and Kikuo Okuyama^3^

^1^Department of Physics, Faculty of Mathematics and Natural Sciences, Institut Teknologi Bandung, Bandung 40132, Indonesia

^2^Research Center for Nanoscience and Nanotechnology, Bandung 40132, Indonesia

^3^Department of Chemical Engineering, Graduate School of Engineering, Hiroshima University, Higashi-Hiroshima 739-8527, Japan

* [ferry@fi.itb.ac.id](mailto:ferry@fi.itb.ac.id)


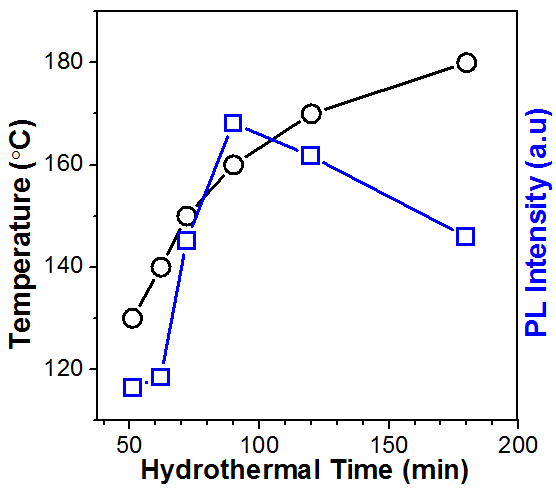


**Figure S1.** Experimental conditions: Temperature profile (circles) of autoclave during synthesis of GQDs, and dependence of PL intensity (square) on hydrothermal time.


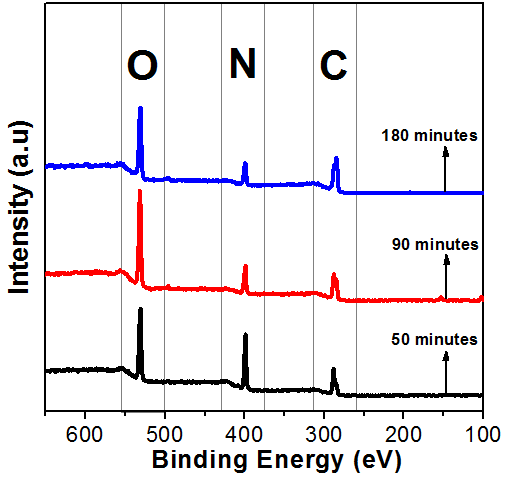


**Figure S2.** Full scan XPS spectra of GQDs with hydrothermal time of 50 min (black line), 90 min (red line), and 180 min (blue line)..


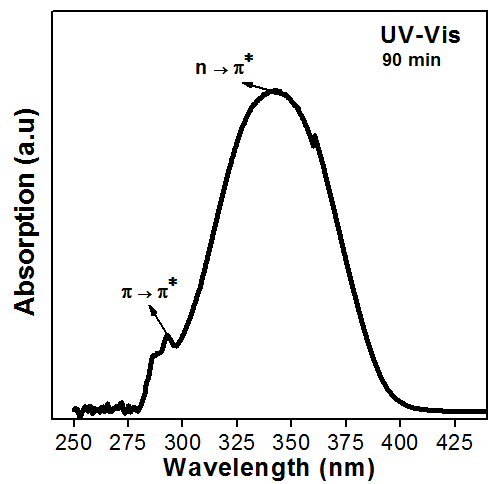


**Figure S3.** UV-Vis absorption of GQDs synthesized by 90 min hydrothermal heating, deconvoluted transition (dashed line) and transition (dotted line).


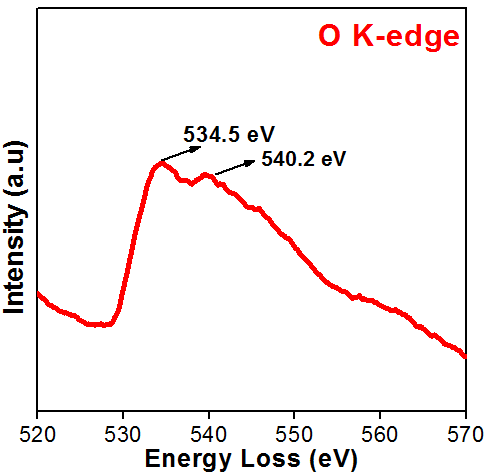


**Figure S4.** EELS spectrum of O K-edge of 90 min GQDs.
